# Individual Differences in the Alignment of Structural and Functional Markers of the V5/MT Complex in Primates

**DOI:** 10.1093/cercor/bhw180

**Published:** 2016-09-19

**Authors:** I. Large, H. Bridge, B. Ahmed, S. Clare, J. Kolasinski, W. W. Lam, K. L. Miller, T. B. Dyrby, A. J. Parker, J. E. T. Smith, G. Daubney, J. Sallet, A. H. Bell, K. Krug

**Affiliations:** 1 Department of Physiology, Anatomy and Genetics, University of Oxford, Oxford, OX1 3PT, UK; 2 FMRIB Centre, Nuffield Department of Clinical Neurosciences, University of Oxford, Oxford, OX3 9DU, UK; 3 Danish Research Centre for Magnetic Resonance, Centre for Functional and Diagnostic Imaging and Research, Copenhagen University Hospital Hvidovre, 2650 Hvidovre, Denmark; 4 Department of Applied Mathematics and Computer Science, Technical University of Denmark, 2800 Kongens Lyngby, Denmark; 5 Department of Experimental Psychology, University of Oxford, Oxford, OX1 3UD, UK; 6 MRC Cognition and Brain Sciences Unit, Cambridge, CB2 7EF, UK

**Keywords:** histology, monkey, MRI, myelination, visual cortex

## Abstract

Extrastriate visual area V5/MT in primates is defined both structurally by myeloarchitecture and functionally by distinct responses to visual motion. Myelination is directly identifiable from postmortem histology but also indirectly by image contrast with structural magnetic resonance imaging (sMRI). First, we compared the identification of V5/MT using both sMRI and histology in Rhesus macaques. A section-by-section comparison of histological slices with in vivo and postmortem sMRI for the same block of cortical tissue showed precise correspondence in localizing heavy myelination for V5/MT and neighboring MST. Thus, sMRI in macaques accurately locates histologically defined myelin within areas known to be motion selective. Second, we investigated the functionally homologous human motion complex (hMT+) using high-resolution in vivo imaging. Humans showed considerable intersubject variability in hMT+ location, when defined with myelin-weighted sMRI signals to reveal structure. When comparing sMRI markers to functional MRI in response to moving stimuli, a region of high myelin signal was generally located within the hMT+ complex. However, there were considerable differences in the alignment of structural and functional markers between individuals. Our results suggest that variation in area identification for hMT+ based on structural and functional markers reflects individual differences in human regional brain architecture.

## Introduction

Much of our understanding of the primate brain derives from its segmentation into areas with distinct structure, function, and connectivity. Over the past century, neuroscientists have attempted to map cerebral cortex through a variety of different criteria (e.g., Brodmann 1909; Vogt and Vogt 1919; Turner and Geyer 2014; Amunts and Zilles 2015). Structural methods include the differential distribution of cell bodies in gray matter (cytoarchitecture, e.g., Brodmann 1909; Eickhoff et al. 2005) or the pattern of myelination within the cortical ribbon (myeloarchitecture, e.g., Gennari 1782; Flechsig 1920; Clark et al. 1992; Barbier et al. 2002; Bridge et al. 2005). The functional organization of cortical areas can, for example, be defined through neurophysiological recordings in monkeys (Daniel and Whitteridge 1961; Zeki 1974; Van Essen et al. 1981) and more recently, noninvasive magnetic resonance imaging (MRI) in humans (Sereno et al. 1995; Engel et al. 1997; Wandell and Winawer 2011). Low resolution of noninvasive methods and individual differences in human functional cortical organization can both limit the accuracy of cortical maps (Zilles and Amunts 2013; Haxby et al. 2014; Laumann et al. 2015).

The primate visual system, with over 30 distinct cortical areas, provides a model system to assess different approaches to segment and map the cerebral cortex (Felleman and Van Essen 1991). Extrastriate visual motion area V5/MT has been intensively studied in both human and macaque, using a combination of different methodologies. In macaque V5/MT, almost all single neurons are selective for direction of motion and many are also selective for binocular disparity (Dubner and Zeki 1971; Maunsell and Van Essen 1983); neurons are assembled in a distinct columnar organization (Albright et al. 1984; DeAngelis and Newsome 1999). Neural activity in V5/MT has been directly linked to the perception of visual motion and depth, both through trial-by-trial correlation with behavioral responses and electrical microstimulation in discrimination tasks for motion and binocular disparity (Salzman et al. 1990; Britten et al. 1996; DeAngelis et al. 1998; Dodd et al. 2001; Krug et al. 2004; Krug et al. 2013; Cicmil and Krug 2015). Macaque area V5/MT has been consistently identified using 1) anatomical location (Dubner and Zeki 1971; Van Essen et al. 2012), 2) myeloarchitecture determined histologically, which shows a dense band of myelin across the lower cortical layers (Lewis and Van Essen 2000), and 3) retinotopic organization and functional specialization determined neurophysiologically and with functional MRI (fMRI) (Zeki 1974; Maunsell and Van Essen 1983; Albright et al. 1984; Kolster et al. 2009).

The putatively homologous region hMT+ in humans was initially identified based on selectivity for direction of motion using positron emission tomography (PET) (Zeki et al. 1991). Using fMRI, hMT+ has also been linked to the processing of visual motion (Huk and Heeger 2002) and depth (Bridge and Parker 2007). Retinotopic mapping of hMT+ with fMRI supports a division into areas V5/MT and MST (Huk et al. 2002; Amano et al. 2009; Kolster et al. 2010). However, postmortem cytoarchitectural studies (Malikovic et al. 2007) as well as retinotopic mapping of large populations (Wang et al. 2015) indicate that the anatomical location of hMT+ is variable in humans. Dense myelination is also visible near the predicted location for human hMT+ (Annese et al. 2005; Bridge et al. 2014). Therefore, visualization of myelin using in vivo human imaging might allow consistent identification of this area from one person to the next.

Using structural MRI (sMRI), Glasser and Van Essen (2011) showed a region of dense myelination in the human occipital lobe in the location of functional and cytoarchitectonic hMT+, based on group average data from different subject populations. Similarly, using fMRI, retinotopically derived probabilistic maps of hMT+ overlapped with average myelin maps extracted from *T*_1_- and *T*_2_-weighted (*T*_1_w and *T*_2_w) sMRI in humans (Abdollahi et al. 2014). By contrast, at the level of individual human subjects, in vivo MRI studies of myelin density in auditory cortex (Dick et al. 2012) indicated variable overlap between these myelination patterns and functionally defined regions; however, functional maps obtained in the same subjects were based on lower resolution data. Sánchez-Panchuelo et al. (2012) investigated the location of a hypointense myelin band in relation to functionally defined hMT+ at high field and found no consistent relationship across their 4 subjects. In order to reliably identify structural changes in the brain that might underlie dysfunction in clinical conditions, we need to understand whether variability in different markers for cortical areas within individual subjects genuinely reflects different cortical architecture or is a limitation due to methodological considerations.

Our aims are 2-fold: first, in the Rhesus macaque, to test the hypothesis that in vivo sMRI correctly identifies cortical areas of high myelin density within the cortical gray matter ribbon by systematically comparing the outcome of in vivo sMRI with postmortem sMRI and “gold standard” histological data from the same animal. The model system we chose is the well-defined extrastriate visual motion area V5/MT. Second, using high-resolution MRI data from individual human subjects, we aim to assess the congruence of structural and functional delineations of hMT+ based respectively on sMRI markers of myelin and 7T fMRI responses to visual motion. We employed two sMRI protocols in humans that had been previously used to investigate myelin density within the cortical gray matter. The first was equivalent to “myelin maps” computed from the ratio of *T*_1_w/*T*_2_w images at 3T (Yoshiura et al. 2000; Sigalovsky et al. 2006; Glasser and Van Essen 2011). The second was a high field (7T) MP2RAGE scan, providing higher image resolution for investigating myeloarchitecture (Dinse et al. 2015). We demonstrate in the Rhesus macaque that sMRI can assess myelin density as defined by histology in individuals. Nevertheless, the comparison of sMRI myelination and functional definitions of the human homolog hMT+ — obtained at high resolution—reveals considerable individual differences in the alignment of structural and functional markers between individuals.

## Materials and Methods

### Rhesus Macaque

#### Animals

Seven Rhesus macaques (*Macaca mulatta*) were used for this study (4 females and 3 males; mean age 8 ± 3.25 years). Four out of 7 animals were involved in both the MRI and the histology study. All animal procedures were carried out in accordance with Home Office (UK) Regulations and European Union guidelines (EU directive 86/609/EEC; EU Directive 2010/63/EU).

#### In Vivo MRI Scans

Five macaques were scanned under general anesthesia (2–3% sevofluorane) in a horizontal 3T MRI scanner with a full-size bore. Anesthetized animals were placed in an MRI-compatible stereotactic frame (Crist Instrument) in sphinx position. For data acquisition, we used a 4-channel, phased-array, radiofrequency (RF) coil in conjunction with a local transmission coil (Windmiller Kolster Scientific). Five *T*_1_w 3D magnetization-prepared rapid gradient echo scans (*T*_1_w MPRAGE; 0.5 × 0.5 × 0.5 mm^3^; TE = 4.04 ms; TR = 2500 ms; flip angle = 8°, 128 slices) and 13 *T*_2_w 3D turbo spin-echo (TSE) scans with variable flip angle (*T*_2_w; 0.5 × 0.5 × 0.5 mm^3^, TE = 3.51 ms, TR = 100 ms, flip angle = 45°, 128 slices) were acquired within the same session (total scan duration ca. 2 h). Scans of the same type were averaged for each animal; the mean image of the *T*_1_w MPRAGE scans was then divided by the mean image of the *T*_2_w scans to create a *T*_1_w/*T*_2_w image, which we refer to as a *T*_1_w/*T*_2_w “myelin-weighted map” (Glasser and Van Essen 2011).

#### Postmortem MRI Scans

Macaques were perfused transcardially with 4% paraformaldehyde in 0.1 M phosphate buffer and the extracted brains were fixed in this solution (see Ahmed et al. 2012 for details). Seven macaque brains were scanned postmortem in a 4.7T preclinical Agilent scanner at the DRCMR in Copenhagen using an ex vivo setup as described by Dyrby et al. (2011). As part of a larger acquisition program, a 2D *T*_2_w spin-echo (SE) MRI sequence using single-line readout was coronally acquired at 0.27 × 0.27 × 0.27 mm^3^ resolution in a slab covering the dorsal superior temporal sulcus (STS) with V5/MT (*T*_2_w) (matrix size = 256 × 256; TE = 19 ms; TR = 2615 ms; 110 slices; no gap; NEX = 36; scan duration = 6.5 h). Repeated measurements were averaged off-line. Since relaxation times change postmortem in *T*_1_w, postmortem *T*_1_w/*T*_2_w images do not provide the same type of contrast as in vivo and, therefore, we only included *T*_2_w postmortem images here.

#### Histology

**Figure 1.**
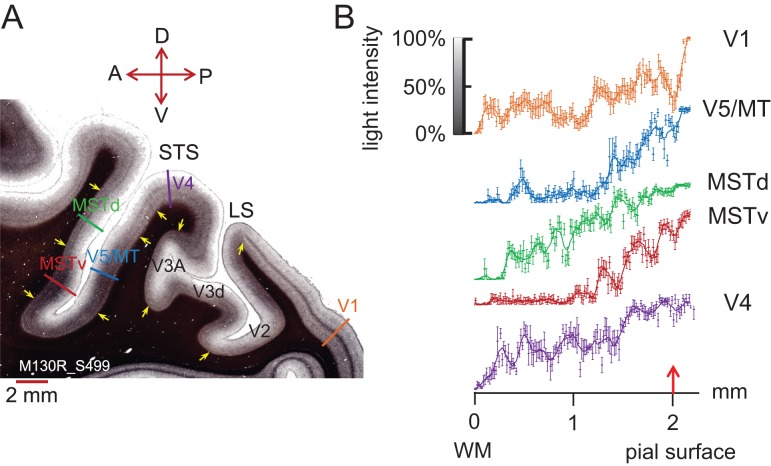
Myelin density across cortical layers. (*A*) A parasagittal section (thickness 30 μm) through occipital cortex from the right hemisphere of a Rhesus macaque (M130R_S499) shows distinct patterns of myelination for visual areas V1, V4, V5/MT, MSTv (ventral MST in our sections), and MSTd (dorsal MST in our sections) after staining with Gallyas. In V1, a dark, dense band of myelin can be seen in layer 4. V4 shows 2 distinct myelin bands, MSTd 1 band. Both areas, V5/MT and parts of MST, show heavy myelination across the lower cortical layers. The colored bars indicate where intensity profiles were measured for panel (B). Yellow arrows give an indication of boundaries between cortical areas based on differences in the myelination pattern. LS, lateral sulcus. Slide orientation: P, posterior; A, anterior; D, dorsal; V - ventral. (*B*) Image intensity profiles of the myelin stain for different visual areas were measured with Neurolucida (Microbrightfield Ltd). Measurements orthogonal to the cortical layers were taken every 15 μm (*n* = 6 measurements per data point). Data points were fitted with a sliding average function (*smooth* rloess function uses weighted linear least squares regression and a second-degree polynomial model, Matlab). Some intensity profiles are distinct, for example, V5/MT and V1. Although at first glance, the image intensity profiles for V5/MT and ventral MST appear similar, they are significantly different from each other when compared quantitatively (2-sample Kolmogorov–Smirnov test, *D* = 0.1987, *P* = 0.0041). Red arrow denotes pial surface. Error bars are ±1 SD.

After perfusion, fixation, and postmortem scanning, brains were cryoprotected in a graded set of 10%, 20%, and, finally, 30% sucrose solutions in 0.1 M phosphate buffer. Brain hemispheres from 6 animals were cut parasagittally on a freezing stage microtome at 50 μm (or 30 μm for M130R in Fig. 1). V5/MT and MST boundaries were determined from myelin-stained sections (Gallyas 1979).

#### Histology Analysis

The histologically defined areas for V5/MT and part of MST (Komatsu and Wurtz 1988; Lewis and Van Essen 2000) were determined by measuring the length of the densely myelinated regions along the dorsoventral pial surface within the dorsal part of STS for each Gallyas-stained parasagittal section (in 1:5, 1:8, or 1:10 series) (with Neurolucida, Microbrightfield Ltd). For V5/MT, the densely myelinated area on the posterior bank was measured, while the densely myelinated area on the anterior bank of the STS was measured for MST. The area of dense myelination was then calculated between successive parasagittal sections of one series with the help of known section spacing and thickness. Total area was aggregated for sections across the mediolateral extent of the densely myelinated area of V5/MT or MST, respectively.

#### In Vivo MRI Analysis

**Figure 2.**
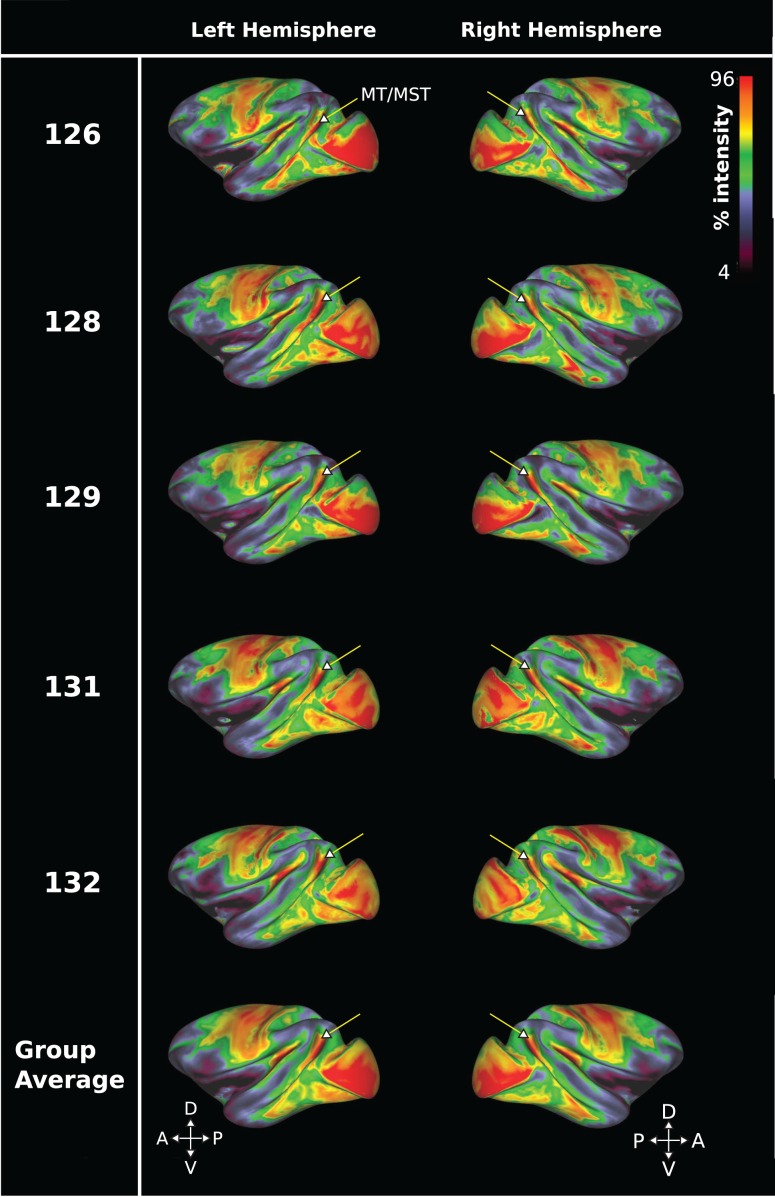
Cortical surface myelin maps from in vivo 3T *T*_1_w/*T*_2_w sMRI data for 5 Rhesus macaques. Data are presented on a color map scale, with red indicating high sMRI signal intensity and therefore regions of greater myelin density. An arrow indicates the approximate anatomical position of V5/MT and MST. Each row displays the left and right hemisphere from a different subject, with the bottom row showing average data. The data show that a high myelin signal delineates a distinct region coinciding with the anatomical location of area V5/MT and MST in the dorsal STS of the Rhesus macaque.

Inflated surfaces were generated from *T*_1_w MPRAGE images, automatically segmented using Freesurfer's recon-all pipeline. *T*_1_w/*T*_2_w images were aligned to the inflated image and converted to GIFTI format, then projected onto the surface using Connectome Workbench (http://www.humanconnectome.org/software/connectome-workbench.html). For visualization, quantitative measures of signal intensity in the *T*_1_w/*T*_2_w images were presented as a color map, with data points outside of 4% and 96% of the full range of intensities set to plateau. For quantitative analysis of cortical areas, maps were imported into MATLAB (The MathWorks) using custom scripts. Those voxels whose signal intensity values lay within the top third of intensity values for each hemisphere (displayed as red in the colored myelin density surface maps in Fig. 2) were defined as “heavily” or “densely myelinated”. The continuous surface area of dense myelination at the anatomical location of the dorsal STS (the stereotactic location of area V5/MT and MST in the Rhesus macaque) was then quantified by calculating the extent of vertices above threshold present in this region in native anatomical space.

### Human

#### Participants

Ten participants (6 males and 4 females) with normal, or corrected-to-normal, vision and with an age range of 24–36 years (mean age 27.8 years) participated in one or more of the scans detailed below. Experiments were undertaken with the understanding and written consent of each participant. All research was conducted in accordance with the guidelines of the Oxford University Central University Research Ethics Committee (CUREC/IDREC), which provided ethical approval for this project (MSD-IDREC-C1-2012-033). Procedures were conducted in accordance with the Declaration of Helsinki (revised 1989).

#### 7T Scan Sessions

All 10 participants were scanned at a high field strength of 7T (Siemens) using a 32-channel receive, 1-channel transmit coil. In the first session, a high-resolution sMRI scan was acquired using the 7T MP2RAGE protocol at 0.7 × 0.7 × 0.7 mm^3^ (7T MP2RAGE) (TE = 3 ms; TR = 5000 ms). Inversion times were set to TI1 = 900 ms and TI2 = 3200 ms, with flip angles of 4° and 5°, respectively. MP2RAGE is a *T*_1_w image that was developed for high field imaging and includes 2 inversion times and thus reduces both the transmit and receive bias fields (Marques et al. 2010). Therefore, no separate *T*_2_w scans were required to reduce the bias field. Three participants underwent a second MP2RAGE scan on a separate day.

Functional MRI at 7T was also conducted with all 10 participants. We acquired echo-planar imaging (EPI) fMRI scans (1 × 1 × 1 mm^3^ isotropic voxel size; 38 slices; TE = 26 ms; TR = 3000 ms; flip angle = 90º; 3 repeats). During the fMRI sessions, participants were instructed to visually fixate on a white dot (diameter = 0.1° of visual angle) displayed centrally on a screen, while a visual stimulus of either coherently moving black and white dot fields on a mid-gray background or checkerboard patterns were presented (screen size = 40º × 34º of visual angle; stimulus dot size = 0.2° of visual angle; dot density = ca. 7/degree).

All 10 participants underwent the first functional scan, which consisted of 3 different block types (each 15 s in duration, and presented 5 times): 1) a full field moving dot stimulus (40° × 34°), 2) a moving dot stimulus limited to a single hemifield, with the edge located 10° from the central fixation point (arranged to cover at least 10 × 34 of the ipsilateral visual field), and 3) a baseline condition of full field static black and white dots. A contrast of the full field moving dot stimulus and the baseline stimulus was used to identify hMT+, and the contrast of hemifield stimulation and baseline was used to identify the ipsilateral activation characteristic of MST (Huk et al. 2002). Eight of the 10 participants took part in a second functional scan session, in which area V5/MT was localized retinotopically with a rotating wedge aperture over a moving checkerboard (5 repeats; after Amano et al., 2009).

#### 3T Scan Sessions

To relate our human data directly to the sMRI obtained from the macaque and to published human sMRI data, we also used another well-established protocol (*T*_1_w/*T*_2_w) to assess myelin-related signals from sMRI at 3T. Three of the 10 participants scanned at 7T were also scanned at 3T (Siemens Verio) using a 32-channel head coil. Two *T*_1_w MPRAGE scans (1 × 1 × 1 mm^3^ isotropic voxel size; TE = 3.77 ms; TR = 2400 ms; Flip angle = 8°), and 3 *T*_2_w 3D TSE scans (1 × 1 × 1 mm^3^ isotropic; TE = 449 ms; TR = 3200 ms) were acquired within the same session. Scans of the same type were averaged; the mean image of the *T*_1_w MPRAGE scans was then divided by the mean image of the *T*_2_w TSE scans to create a *T*_1_w/*T*_2_w image, after the methods of Glasser and Van Essen (2011).

#### Structural Analysis: 7T MP2RAGE

For each of the 10 individuals scanned at 7T, the structural MP2RAGE scan was reconstructed using the Freesurfer recon-all pipeline to create a cortical surface. The same MP2RAGE scan for each participant was brain extracted using FSL-BET (Smith 2002), after which any remaining non-brain structures were removed by hand in Freeview, then overlaid onto the individual inflated hemispheric surface using Freesurfer's mri_vol2surf command (Dale et al. 1999; Reuter et al. 2012). In order to select the appropriate cortical depth on which to center the signal sampling (using the “projfrac” tag of the mri_vol2surf function), multiple surface signal intensity images were generated for each individual hemisphere, varying from 0 to 1 (with steps of 0.1) in their cortical thickness fraction. A value of 0.5, for example, would sample in the middle of the cortical thickness. For 9 out of the 10 participants, the value with the greatest mean intensity was taken at a cortical thickness fraction of 0.6, indicating the strongest “myelin-weighted signal” across cortex. In order to sample consistently across hemispheres, this cortical thickness fraction was used throughout to center the “myelin-weighted maps.” 7T data were resampled into the 1 mm space in order to facilitate the comparison between different types of MRI scans. Thresholding each hemisphere overlay at +1 SEM (standard error of mean) above the mean intensity produced a high signal map across the unfolded cortical surface taken to indicate heavy myelination (defined as 7T MP2RAGE “myelin-weighted map”). For the generation of myelin-weighted maps based on 7T MP2RAGE scans, the temporal lobe of each hemisphere was masked out due to a known artifact resulting in falsely high intensities in this area at 7T (Vaughan et al. 2001), which could bias the intensity threshold.

We statistically compared, in native space, two 7T MP2RAGE scans acquired for the same subject but in separate scan sessions on different days (3 participants). The 7T MP2RAGE scans for each participant were segmented separately using FSL-FAST (Zhang et al. 2001) and then imported into MATLAB for the analysis of signal correspondence using custom scripts. Signal correspondence was compared either across the entire cortex or within a region of interest (ROI) defined for the visual motion complex hMT+ based on the fMRI responses to full field motion (see below). To calculate percentage correspondence between 2 scans across the entire cortex (whole cortex correspondence), myelin-weighted maps were first established by thresholding each of the 2 scans to be compared at mean intensity +1 SEM (as above) and then binarizing the result into voxels above threshold (“myelin signal”) and below to create a mask. The number of overlapping voxels from the 2 masks was then divided by the combination of voxels from the 2 masks (counting the overlapping voxels only once). Correspondence between 2 scans within a specific ROI (ROI correspondence) was similarly calculated: first the 2 scans to be compared were thresholded and binarized (as above). The number of overlapping signal voxels within the ROI was then divided by the total number of thresholded voxels present within the ROI.

#### Structural Analysis: 3T *T*_1_w/*T*_2_w

For establishing myelin-weighted maps from the 3T data, the same analysis procedure was employed as described above for 7T MP2RAGE (but without the temporal lobe masking). However, the inflation generated in Freesurfer was from one structural *T*_1_w MPRAGE scan, and the myelination surface map was generated from the averaged *T*_1_w/*T*_2_w image. To facilitate direct comparisons, the *T*_1_w/*T*_2_w surface map was also aligned to the individual's 7T structural scan via FSL-FLIRT with 7 DOF. The registration was applied within Freesurfer in order to produce a *T*_1_w/*T*_2_w surface map in the space of the scan acquired at 7T (referred to as 3T *T*_1_w/*T*_2_w “myelin-weighted map”).

To compare the correspondence between the myelin-weighted maps derived from 7T MP2RAGE and 3T *T*_1_w/*T*_2_w images, the whole cortex correspondence and within ROI correspondence for high signal voxels were analyzed as above.

#### Functional Analysis of 7T fMRI

Data from high-resolution fMRI scans (obtained at 7T; 10 participants) were analyzed in FSL-FEAT and aligned to the structural scans using FSL-FLIRT (Jenkinson et al. 2002, 2012). Data were linearly registered to a whole-head EPI with the same slice prescription (3 DOF translation only), then to the high-resolution structural MP2RAGE (BBR algorithm; Greve and Fischl 2009), also obtained at 7T. For each of the 3 functional scan repeats, a general linear model was used to model the activation for each of the moving stimulus types compared with the static dots within FSL-FEAT, and thresholded at *z* > 2.3, with a cluster threshold of *p* = 0.05. This allowed us to assess the quality of the data and registrations and examine the level of subject movement. The unthresholded data for the 3 individual runs were then combined using a fixed effects model, with a *z*-threshold of >2.3 and cluster threshold of *p* = 0.05. A continuous area of activation in response to the full field dot stimulus near the putative anatomical location of area hMT+ was taken as the hMT+ complex (or the hMT+ ROI). Within the hMT+ ROI, activation due to ipsilateral visual motion stimulation was assigned to area MST. This activation sometimes formed a single cluster that extended outside of hMT+, in which case the entire cluster was included. Activity within area hMT+ that showed a contralateral retinotopic map was assigned to area V5/MT. Polar and eccentric retinotopy were analyzed within the Freesurfer retinotopy pipeline (8 participants). Phase-encoded data were analyzed via Fourier analysis, which results in a phase and amplitude value at each voxel. These were averaged over multiple within-individual repeat scans to provide a polar retinotopic map. ROIs were considered significant if the coherence of the response to the stimulus exceeded 0.25. For 2 human subjects (004 and 007), ipsilateral activity was also evident within the retinotopically active region—this activity was excluded from the definition of V5/MT but included in area MST. All functional images were corrected for head motion in preprocessing, and to preserve the spatial resolution, no smoothing was applied. Processed data were imported into Freesurfer, then registered and overlaid onto the cortical surface. The quality of registration was assessed by eye via the Freesurfer tkmedit tool and found in all cases to be well matched.

To determine group responses, a higher level analysis was conducted for full field and ipsilateral data across all 10 subjects with a mixed effects (FLAME 1 + 2) model within FSL. The output of this analysis was rendered onto an inflated average structural scan within Freesurfer, using the same procedure as for the individual subject data. The group average location of retinotopic area V5/MT was generated by binarizing the V5/MT mask from each of the 8 individuals who took part in the second functional scan. For left and right hemisphere separately, we summed the 8 V5/MT masks and then thresholded this summed image to give the region in which ≥50% of individuals showed overlapping activation. This thresholded group definition was then also overlaid onto the average structural surface within Freesurfer.

#### Comparison Between Structural and Functional Markers

Assessment of the correspondence between 7T MP2RAGE or *T*_1_w/*T*_2_w myelin-weighted maps derived from sMRI and functional definitions based on fMRI was achieved by importing the functional and structural data for individuals or the group average into MATLAB for further analysis. We calculated the percentage of voxels that were both within functionally defined area hMT+ and that also had above threshold intensity for the sMRI scan (taken as indication of dense myelin). These percentages were also calculated for functionally defined for areas V5/MT and MST.

#### Threshold Analysis

To investigate whether threshold levels for the analysis of sMRI and fMRI data affected our results, thresholds for structural and functional images were systematically varied. For the 7T MP2RAGE myelin-weighted maps, 3 further thresholds were examined in addition to the standard threshold of mean intensity +1 SEM. These thresholds were 1) exactly at the mean intensity—termed “very low threshold”; 2) at mean intensity +0.5 SEM as a “low threshold”; and 3) mean intensity +1.5 SEM as a “high threshold”. The resultant area definitions were compared with the original functional definitions obtained for *z* > 2.3. Functional definitions were also varied systematically, examining 3 thresholds in addition to the original *z*-threshold of >2.3. 1) As a “very low threshold,” we used *z* > 1.3, 2) as a “low threshold” *z* > 1.8, and 3) as the “high threshold” *z* > 2.8. These resultant functional definitions were then compared with the original myelin-weighted map obtained at mean intensity +1 SEM.

## Results

### Comparing Myelin Maps Obtained from Histology and sMRI in the Rhesus Monkey

Cortical myelin can be visualized with a silver Gallyas stain in postmortem cortical sections (Gallyas 1979). In the Rhesus macaque, Gallyas-stained, histological sections through occipital cortex show a thick band of dark myelination across deeper cortical layers on the posterior bank of the STS, which delineates visual area V5/MT, whereas visual areas V1 or V4 can be delineated by the presence of 1 or 2 distinct thin bands on a lighter background as shown in Figure 1*A*. Cortical area MST on the anterior bank of the STS comprises up to 3 zones with distinct patterns of cortical myelination (Komatsu and Wurtz 1988; Lewis and Van Essen 2000): The parasagittal section in Figure 1 shows a thick band of heavy myelination on the anterior bank of the STS across the lower cortical layers, which may overlap with MSTda of Lewis and Van Essen (2000). We also identify a lighter region with a single, thin band of myelin more dorsally. These patterns can be assessed quantitatively by measuring the intensity profile through the gray matter at different cortical locations (Fig. 1*B*). The colored bars in Figure 1*A* indicate the approximate path over which the intensity profiles were measured for different visual cortical areas to produce the graph in Figure 1*B*. For further analysis, we use the 2 areas with distinctly dense myelin in our macaque brains: visual area V5/MT and the more ventral subdivision of MST on the anterior bank, termed ventral MST (MSTv) here.

**Figure 3.**
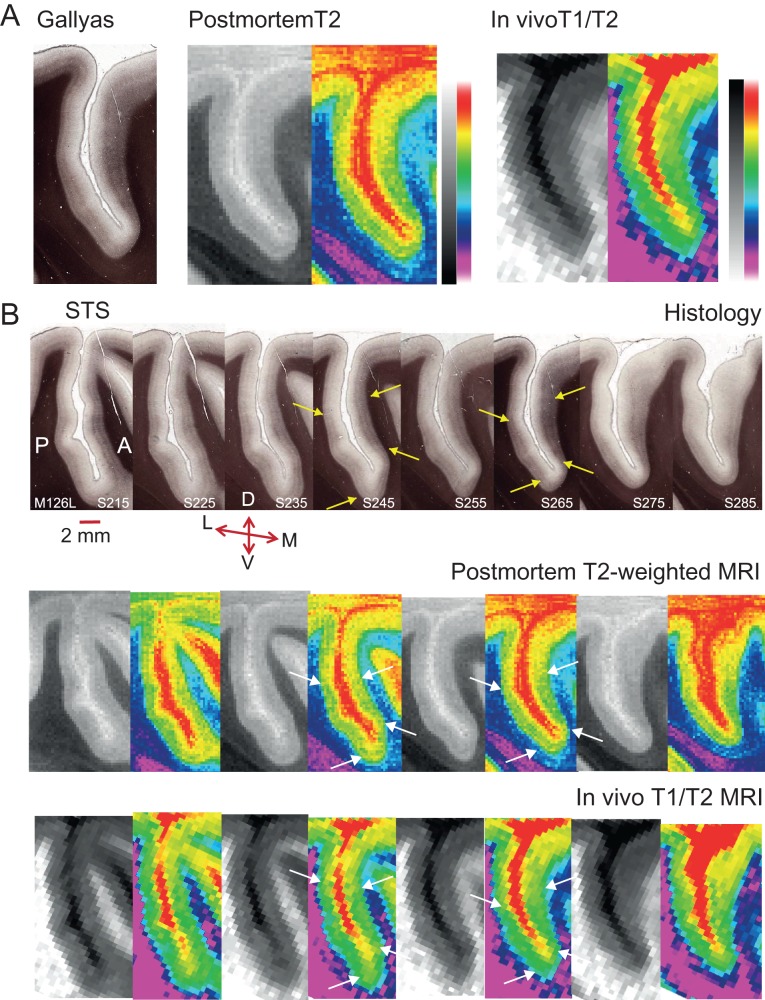
Slice-by-slice comparison of histological sections and matched sMRI for the Rhesus macaque. (*A*) A Gallyas-stained parasagittal section showing the dorsal part of the STS from the left hemisphere of 1 macaque (M126L, S265) was matched to the equivalent parasagittal sections obtained previously from a postmortem scan of the same intact, fixed brain and to MRI obtained from the same brain in vivo. The histological section is 50 μm thick: dense cortical myelin shows up as dark brown, particularly in the lower cortical layers in the histological sections. In the postmortem *T*_2_w MRI scans, this is evident in dark regions relative to surrounding cortical tissue, and as lighter voxels relative to surrounding cortical tissue for the in vivo *T*_1_w/*T*_2_w images. To enhance visibility, the same sMRI images were pseudo-colored by converting to a color gradient (Adobe Photoshop; gradient map “transparent rainbow”; postmortem images were contrast inverted to yield the same color scale). (*B*) Shown is a 1 in 10 series of 50 μm parasagittal sections with a separation of 500 μm between consecutive histological sections for the same hemisphere (M126L) as in (*A*). The series was matched to the equivalent sMRI slices obtained postmortem or in vivo from the same block of cortical tissue. Yellow/white arrows indicate borders of dense myelination for extrastriate visual area V5/MT on the posterior bank of the STS, and for parts of MST on the anterior bank across all 3 methods for 2 example sections. Every second MRI section was replaced with a pseudo-colored version (see *A*.). The original MRI sections from M126L and from a further example brain are available online as Supplementary Figure S1. Series orientation: lateral (L) to medial (M). Section orientation: D, dorsal; V, ventral; P, posterior; A, anterior.

While such banding patterns within the cortical ribbon can be visualized histologically, sMRI voxels usually span multiple cortical layers (but see also Dinse et al. 2015). If sMRI contrast is sensitive to structural differences associated with cortical myelin, the average signal in these voxels should still differ according to the underlying myelin density. To test this directly, histological data collected from 3 Rhesus monkeys were compared with postmortem and in vivo sMRI scans previously obtained from the same brain. Fifty-micrometer-thick parasagittal brain sections stained with Gallyas were matched with sMRI slices in terms of the mediolateral position in the brain and both taken at 500 μm intervals from the same hemisphere (Fig. 3 and Supplementary Fig. S1). In the histological sections, myelin was stained dark brown. In postmortem *T*_2_w scans, areas of dense myelination, like white matter, show decreased signal intensity. In both types of sections, darker regions across the deeper layers of cortex on either side of the sulcus show dense myelination for V5/MT and MST (e.g., see pairs of yellow/white arrows in Fig. 3*B*). The same cortical regions at the border with white matter in the matched in vivo *T*_1_w/*T*_2_w MRI slices appear lighter, because *T*_1_ shows higher signal intensities for dense myelination. The corresponding changes in pattern of signal intensity across equivalent sections demonstrate that the postmortem and in vivo sMRI signals can detect qualitative changes in cortex equivalent to the staining of myelin in the histological sections.

To visualize the entirety of the cortical region, we mapped the in vivo *T*_1_w/*T*_2_w sMRI data onto the inflated cortical surface in 5 macaques for each brain individually and as a group average. For both the individual hemispheres and group average data, we can identify a distinct region of high signal around the dorsal STS in these *T*_1_w/*T*_2_w myelin-weighted maps (Fig. 2). This matches the sulcal location of V5/MT and MST, which are known from histological studies in the Rhesus monkey to be densely myelinated (see also Figs 1 and 3). However, unlike for the parasagittal histology data, where the fundus of the STS appears to provide a border with apparent changes in myelination density, these *T*_1_w/*T*_2_w myelin-weighted maps on the inflated cortical surface appeared to delineate only one extended area of dense myelination in this region. Therefore, the distinction between cortical areas V5/MT and MST was difficult when using in vivo sMRI data alone. When individual *T*_1_w/*T*_2_w myelin-weighted maps were projected onto a standard brain, a pairwise comparison in native space revealed a high degree of consistency from subject-to-subject for the location of this distinct region of heavy myelination in the dorsal STS (mean overlap left hemisphere: 85.1%, SD = ±6.9; right hemisphere: 87.7%, SD = ±5.8).

We measured the mean surface area of this region of distinct myelination (top third of image intensity values) obtained with in vivo sMRI in native anatomical space as 82.4 mm^2^ (SD = ±8.9; *n* = 10 hemispheres). This number is larger than published average measurements for histological V5/MT in *M. mulatta* or *M. fasicularis*, which range between 33 and 80 mm^2^ (Van Essen et al. 1981; Maunsell and Van Essen 1987; Sincich and Horton 2003). We compared the size of the area of myelination obtained in vivo to measurements we took from the histological sections stained with Gallyas of 4 of the same and 2 additional brains (Table 1). Our average histological measurement of area V5/MT was 35 mm^2^ (SD = ±7.2; *n* = 7 hemispheres) and for area MST, 41 mm^2^ (SD = ±4.5; *n* = 7 hemispheres). Thus, the cortical surface area detected for in vivo *T*_1_w/*T*_2_w myelin-weighted maps was close to that measured from histological sections for V5/MT and MST combined (76 mm^2^). Both, the relatively large voxel size and the presence of voxels that only overlap partially with layers of dense myelination are likely to contribute to differences. Across the 4 individual hemispheres with both in vivo sMRI and a corresponding histological myelin map, there was a strong correlation between the sizes of the densely myelinated areas measured for the complex formed by V5/MT and MST with the 2 methods (*r* = 0.98; *P* < 0.05; *n* = 4 hemispheres from 4 animals). This suggests that there is a consistent relationship between the 2 methods of identifying myelination, but the sMRI technique is not able to distinguish V5/MT from MST.

**Table 1:** Myelin surface area in the Rhesus macaque for area MT + MST from MRI and histology

Subject	sMRI lh (mm²)	Histology lh (mm²)	sMRI rh (mm²)	Histology rh (mm²)
126	71.4	66	79.1	—
127	—	71.7	—	—
128	94.7	90.3	86.2	—
129	88.5	81.6	83.6	—
130	—	68.8	—	65.1
131	94.1	89.3	82.3	—
132	69.2	—	74.6	—
Mean (SD)	83.6 (±12.4)	78 (±10.6)	81.2 (±4.5)	

This table compares measurements of myelin surface area for MT + MST from 7 Rhesus macaques. Measurements were taken from sMRI or histology and are in square millimeters. lh, left hemisphere; rh, right hemisphere.

### Matching Functional Localizers to Myelin Maps in Humans

**Figure 4.**
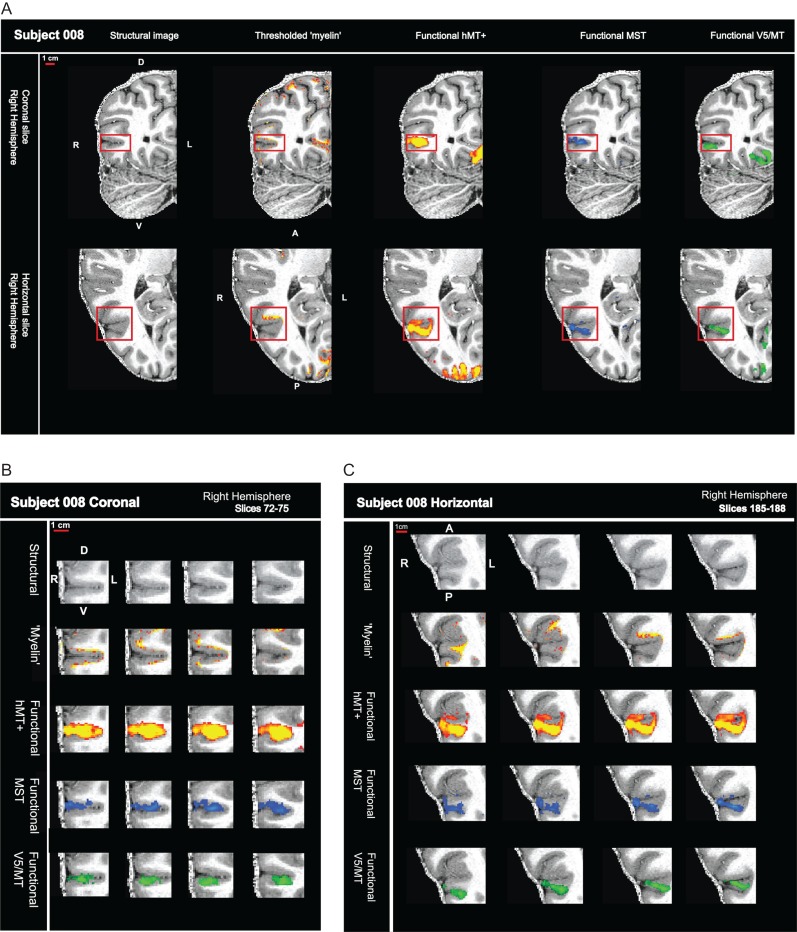
High field fMRI and sMRI in humans localizes signals to the cortical ribbon. (*A*) sMRI (7T MP2RAGE) and fMRI (7T) data are shown for one hemisphere—in a coronal and horizontal slice. The sequence of images show, from left to right, the structural scan first on its own, then either with the myelin-weighted map overlaid (red-yellow scale; threshold: mean intensity +1 SEM) or with functional activation for full field motion (red-yellow scale; threshold: *z* > 2.3; cluster threshold: *p* = 0.05), with functional activation for ipsilateral motion (blue; threshold: *z* > 2.3; cluster threshold: *p* = 0.05) and with the contralateral polar retinotopic activation (green). Red box contains the visual motion area hMT+. (*B*) Close-up view of sequential coronal slices for red box area in (*A*). (*C*) Close-up view of sequential horizontal slices for red box area in (*A*).

In the previous section, we showed that in vivo sMRI protocols are sensitive to structural differences associated with cortical myelin in individual Rhesus macaques*. *Postmortem histological examination of human cortex has demonstrated a region of myelination associated with the approximate sulcal location of area hMT+ (Annese et al. 2005). Here, we examine whether a heavily myelinated region can be reliably detected with in vivo sMRI in the same region in the human brain of individual subjects. This area is compared with the standard functional definitions of the hMT+ complex, the human homologue of areas V5/MT and MST in the Rhesus monkey, in the same subjects. To achieve high spatial resolution, we acquired some of the sMRI and all of the fMRI data at 7T magnetic field strength. Structural myelin-weighted signals and functional activations were well localized within the cortical ribbon (Fig. 4). However, while fMRI activations spanned the cortical layers as expected, we found myelin-weighted sMRI signals around hMT+ predominantly near the gray matter/white matter boundary. Often, nearby cortical areas also showed indications of high myelin-weighted signal.

#### Functional Definitions of hMT+, V5/MT, and MST

**Figure 5.**
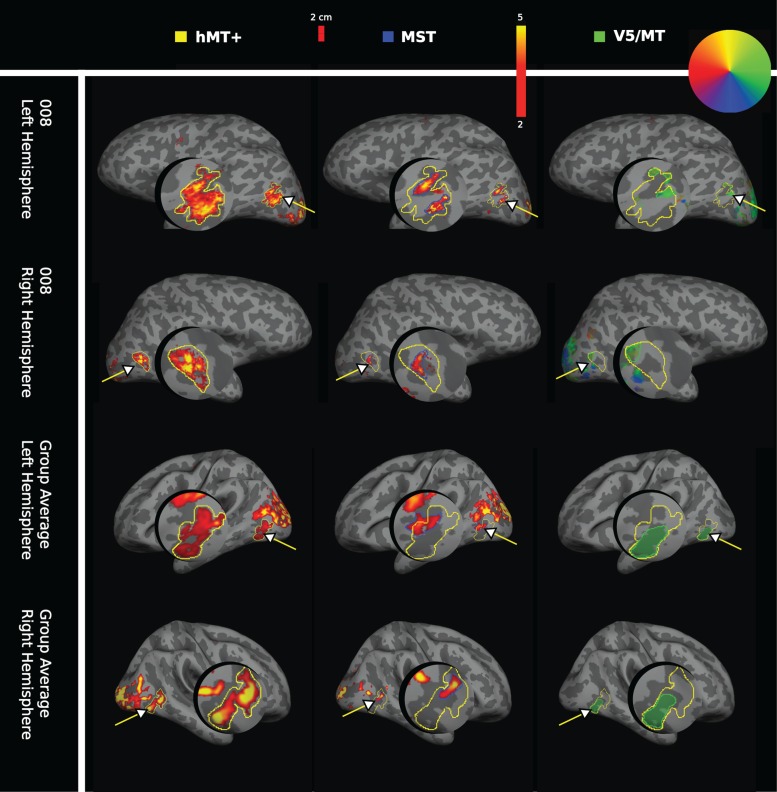
Individual and average functional activation for visual areas hMT+, MST, and V5/MT in humans. Functional activation for visual areas hMT+, MST, and V5/MT (arrows) are shown on the inflated cortex including a magnified cutout of the area of interest. The first column shows the functional activations for area hMT+ to a full field moving dot stimulus (yellow outline), the second column the ipsilateral activation for area MST (blue outline), both shown on a red-yellow signal intensity scale. The third column shows contralateral polar retinotopic activation for area V5/MT (green outline), color-coded according to a retinotopic color wheel for the brain of participant 008. The group average masks for area V5/MT were colored green for consistency. Activation patterns are presented for the left and right hemispheres, for subject 008 (first and second row) and for the group average (*n* = 10 subjects for hMT+ and for MST; *n* = 8 subjects for V5/MT; third and fourth row). For the retinotopy used to localize V5/MT, the group average shows the average of the individual masks for V5/MT activation.

Functional localization of hMT+ was performed using full field moving dots compared with stationary dots. The region corresponding to area MST was identified as a subregion of this motion-sensitive cortical area, using a moving dot stimulus restricted to the ipsilateral hemifield. Retinotopic mapping for the contralateral visual hemifield was used to map V5/MT functionally. The respective areas activated are shown on the inflated cortical surface for an individual participant and the group average (Fig. 5). Consistent with earlier reports (Huk et al. 2002), we found that functional MST was generally anterior to V5/MT, but functional MST would sometimes extend outside of the region localized as hMT+. Four out of 16 hemispheres showed some degree of overlap between functional definitions of V5/MT and MST as previously shown (Huk et al. 2002, 4/20 hemsipheres). In these cases, ipsilateral activation defined as MST was excluded from the definition of V5/MT for the purpose of further analysis.

#### Comparing Myelin-Weighted Maps from 7T MP2RAGE and 3T *T*_1_w/*T*_2_w Structural Images

**Figure 6.**
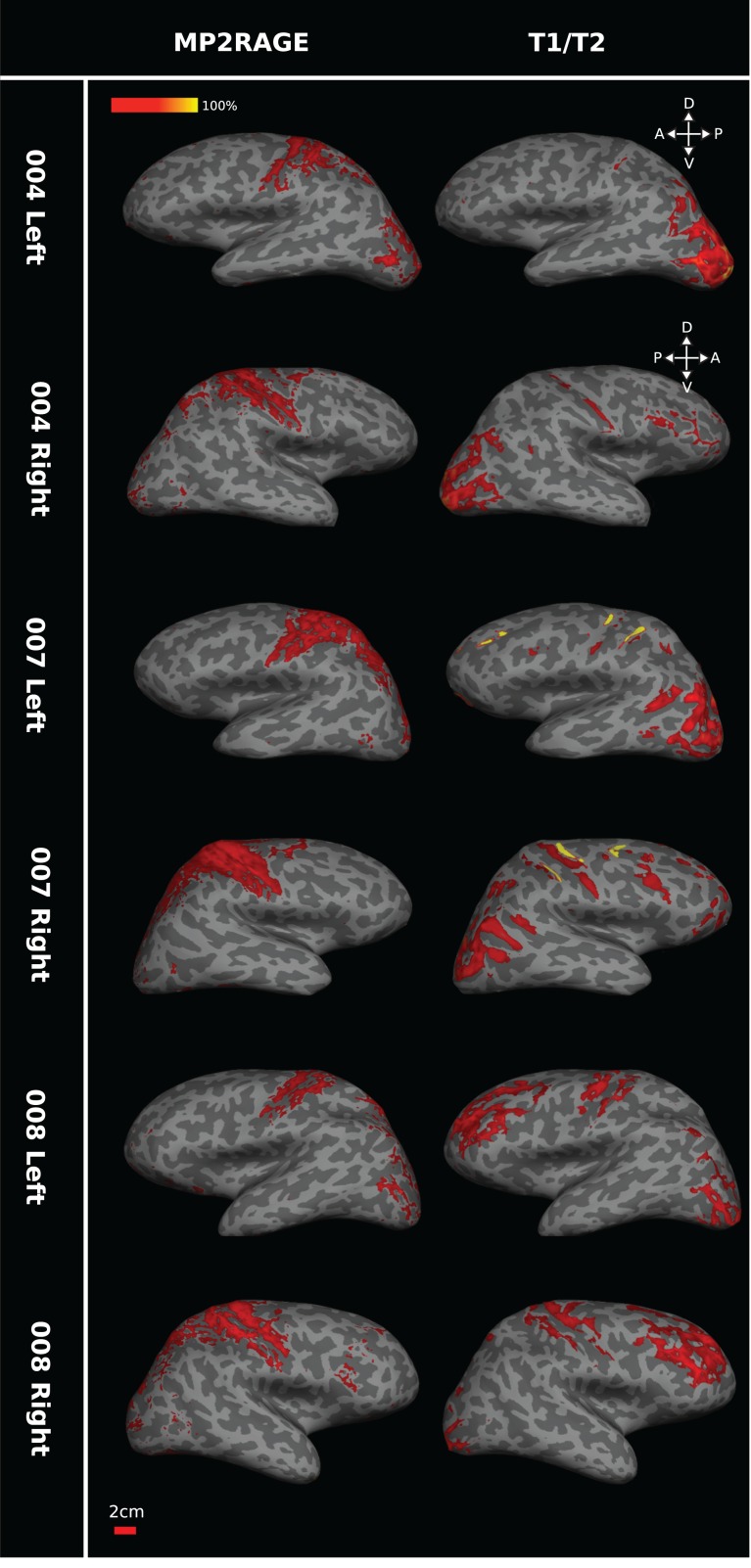
Comparing 7T MP2RAGE and 3T *T*_1_w/*T*_2_w scans for myelin in humans. The pair of images in each row shows the myelin maps obtained for one hemisphere of the same individual participant based on the MP2RAGE images at 7T (left) or on *T*_1_w/*T*_2_w at 3T (right). While 7T MP2RAGE appears more sensitive to central sulcus myelin and 3T *T*_1_w/*T*_2_w images to occipital myelin and despite some individual variation, the general pattern in these images indicates consistency of high sMRI signal in regions associated with dense myelination (central sulcus, V1, hMT+). Data for each of the 3 participants (004, 007, and 008) are presented on each individual's inflated surface generated from 7T structural scans. Brain orientation: D, dorsal; V, ventral; A, anterior; P, posterior.

For 3 participants, sMRI data were derived from both MP2RAGE at 7T field strength and *T*_1_w/*T*_2_w images at 3T field strength; both these sMRI protocols can detect structural differences associated with cortical myelin. Heavily myelinated regions were defined as those with a signal intensity of +1 SEM above the mean (Fig. 6). A subject-by-subject comparison between these 7T MP2RAGE and 3T *T*_1_w/*T*_2_w sMRI myelin-weighted maps on the cortical surface showed consistent regions of high signal intensity in locations predicted from the known pattern of dense myelination, for example, around the central sulcus and in the occipital lobe around V1 and hMT+. However, there were regional differences in sensitivity between the two scanning protocols. MP2RAGE generated stronger signals around the central sulcus, while *T*_1_w/*T*_2_w showed greater intensities around the occipital pole. The correspondence between the 2 different maps across the cortical surface for individuals ranged from 38% to 87% (Table 2). We predicted chance levels of 2–6% correspondence by shuffling the data from these scans to produce images of randomly placed intensities and assessing the correspondence levels between these shuffled images. The measured whole cortex correspondence between the 2 types of scan was significantly better than chance (permutation test; number of repeats = 1000; *P* < 0.01).

**Table 2:** Correspondence between myelin-weighted maps from 7T MP2RAGE and 3T *T*_1_w/*T*_2_w images in humans

Subject	Whole cortex	hMT+ lh	hMT+ rh
4	38.2%	79.9%	18.8%
7	54.0%	45.6%	13.5%
8	86.9%	87.5%	14.2%
Group Average	89.2%	68.1%	12.6%

This table summarizes the percentage correspondence between the 2 myelin-weighted maps obtained from 7T MP2RAGE and 3T *T*_1_w/*T*_2_w hemisphere-by-hemisphere for 3 human participants and for the group average. lh, left hemisphere; rh, right hemisphere.

Inter-scan type correspondence for the left hemisphere ROI for hMT+ alone was high, ranging from 46% to 88% between individuals (Table 2). But the inter-scan correspondence was lower, at 14–19% for the right hemisphere hMT+ ROI. As with whole cortex correspondence above, shuffled data within the ROI of functionally defined hMT+ showed that the level of inter-scan type correspondence was significantly above chance (range of 1–8%; permutation test; *n* = 1000; *P* < 0.01). Across all individuals, inter-scan type correspondence was higher for left hMT+ than for right hMT+, and overall, the total area above intensity threshold for the myelin-weighted map was smaller in the right hemispheres. As the threshold for each hemisphere was computed separately, this result is unlikely to be the product of incorrect choice of threshold reference points.

To determine the between-scan reliability of the sMRI data derived from the 7T MP2RAGE scans, 3 participants were re-scanned at a later date with the same scan protocol and the percentage overlap between the high signal intensity regions, which define the 7T MP2RAGE myelin-weighted map, was calculated. Across individuals, overlap ranged from 98.1% to 99.8% for the whole cortex and in the region of hMT+ for each hemisphere (Table 3), showing high reproducibility of sMRI results when the same scanning protocol was used in the same individual.

**Table 3:** Correspondence between repeated 7T MP2RAGE scans in humans

Subject	Whole cortex	hMT+ lh	hMT+ rh
7	99.8%	98.1%	98.2%
21	99.2%	99.1%	99.5%
22	99.8%	99.6%	99.1%

Three participants were re-scanned with the same 7T MP2RAGE protocol. The table shows high levels of correspondence between the 7T MP2RAGE myelin-weighted maps obtained on different days for the same individual. The myelin-weighted maps for the region of hMT+ obtained for each of the 3 participants did not significantly differ between repeated scans (paired *t*-test; *P* = 0.62, *n* = 3501 voxels; *P* = 0.88, *n* = 3274 voxels; *P* = 0.83, *n* = 2890 voxels). lh, left hemisphere; rh, right hemisphere.

#### Comparison of Structural and Functional Data

**Figure 7.**
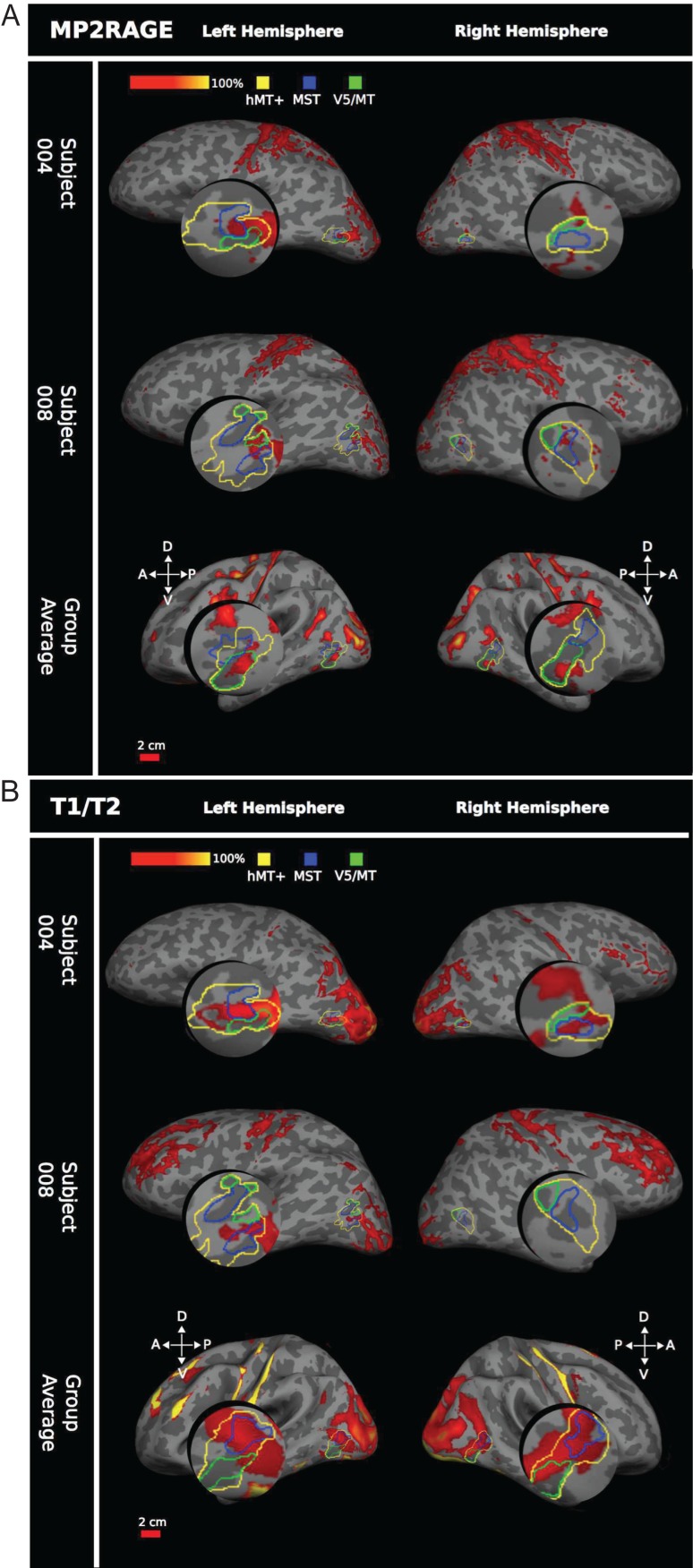
Comparing myelin intensity with functional hMT+ in humans. (*A*) 7T MP2RAGE myelin, 2 example participants and group average (*n* = 10 participants). Functional hMT+ is outlined in yellow, functional MST in blue, and retinotopically defined V5/MT in green. Myelin dense areas were calculated as +1 SEM above the mean intensity of the 7T MP2RAGE image, shown on a red-yellow scale of intensity values. In all hemispheres, a region of dense myelination is associated with area hMT+, MST, or V5/MT. However, there is no consistent association across individuals. All data are presented on the individual subject's inflated cortical surface generated from a 7T structural scan, or the average generated surface (for the average data). (*B*) Human 3T *T*_1_w/*T*_2_w myelin shows 2 example subjects plus group average (*n* = 3 participants). Similar measurements and same conventions as in (*A*), but here the myelin maps were generated from the 3T *T*_1_w/*T*_2_w scan.

When the human group average myelin-weighted maps were overlaid on an average cortical surface and directly compared with group averaged functional definitions obtained from the same subjects, a patch indicating dense myelination showed a clear association with functionally defined area hMT+ in both hemispheres and for both structural scan types (Fig. 7). This was the case for both the 7T MP2RAGE (*n* = 10 participants) and the 3T *T*_1_w/*T*_2_w (*n* = 3 participants) scans. However, the group average 7T MP2RAGE data indicated a smaller, more distinct, area of dense myelination, corresponding almost exclusively to area V5/MT as defined by retinotopy (Fig. 7*A*). The 3T *T*_1_w/*T*_2_w data showed a larger area of myelination spreading beyond hMT+. This area encompassed most of MST and parts of area V5/MT (Fig. 7*B*), which is confirmed by the quantitative analysis of the overlap between myelin signal and functional definitions of these areas (Tables 4 and 5).

**Table 4:** Overlap of 7T MP2RAGE myelin with functionally localized hMT+, V5/MT, and MST in humans for individual hemispheres and group averages

Subject	Hemisphere	%hMT+ myelinated	%V5/MT myelinated	%MST myelinated
1	lh	3.2%	0.0%	6.8%
	rh	0.0%	0.0%	0.0%
2	lh	0.0%	—	0.0%
	rh	3.1%	—	14.9%
3	lh	17.9%	22.0%	0.0%
	rh	1.9%	0.0%	39.7%
4	lh	31.0%	12.1%	50.0%
	rh	4.5%	13.7%	0.2%
7	lh	7.3%	9.9%	12.2%
	rh	14.2%	20.3%	33.2%
8	lh	23.7%	44.5%	6.1%
	rh	11.3%	1.0%	22.3%
11	lh	13.3%	—	2.0%
	rh	1.8%	—	12.6%
21	lh	12.1%	20.6%	2.3%
	rh	18.5%	11.4%	7.6%
22	lh	6.1%	1.4%	0.0%
	rh	12.2%	3.1%	4.1%
38	lh	34.2%	20.0%	7.4%
	rh	15.3%	9.3%	2.2%
Group	lh	42.3%	58.1%	1.9%
Average	rh	18.0%	32.2%	0.1%

This table summarizes measurements of the percentage of functionally defined cortical regions hMT+, V5/MT, and MST (in response to visual motion stimuli, see Fig. 5) that is also indicated as heavily myelinated (based on 7T MP2RAGE myelin-weighted maps). Individual participants and group averages are shows. Missing values for V5/MT are for 2 participants for whom a retinotopy scan was not obtained. lh, left hemisphere; rh, right hemisphere.

**Table 5:** Overlap of 3T *T*_1_w/*T*_2_w myelin with functionally localized hMT+, V5/MT, and MST in humans for individual hemispheres and group average

Subject	Hemisphere	%hMT+ myelinated	%V5MT myelinated	%MST myelinated
4	lh	67.2%	31.9%	67.0%
	rh	50.3%	31.2%	79.2%
7	lh	15.2%	24.2%	28.9%
	rh	9.9%	1.3%	51.0%
8	lh	18.0%	2.1%	16.4%
	rh	1.8%	0.9%	3.4%
Group	lh	59.8%	27.7%	92.4%
Average	rh	85.6%	40.8%	94.1%

This table summarizes the measurements of the percentage of functionally defined cortical regions hMT+, V5/MT, and MST (in response to visual motion stimuli, see Fig. 5) that is also indicated as heavily myelinated (based on 3T *T*_1_w/*T*_2_w myelin-weighted maps). lh, left hemisphere; rh, right hemisphere.

While the group data showed good correspondence between the functional definition of hMT+ and a region of heavy myelination, this association was less consistent across individual hemispheres, especially for the 7T MP2RAGE images. Visual inspection of 3T *T*_1_w/*T*_2_w scans (Fig. 7*B*) always showed a region of high-intensity signal associated with the functional location of area hMT+. However, this region was not exclusively aligned with either functional MST or retinotopic V5/MT, although there appeared to be a somewhat greater overlap with area MST and association with the border region between V5/MT and MST across the 3 subjects. The individual 7T MP2RAGE scans were more variable in terms of the strength of the myelin signal detected near hMT+, but generally followed a similar trend to the 3T *T*_1_w/*T*_2_w results—a smaller myelin region associated with hMT+ usually in a similar location. There was no consistent alignment with either MST or V5/MT across the individual subjects (Fig. 7*A*).

**Figure 8.**
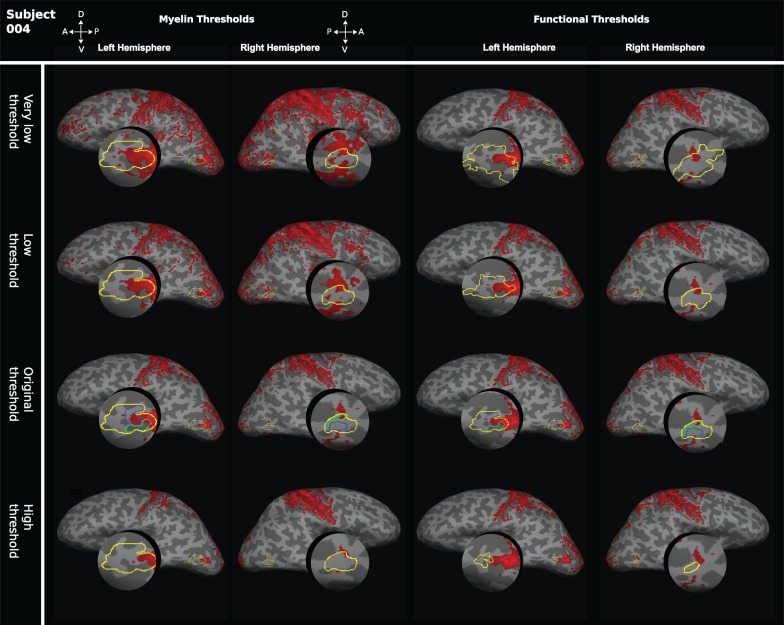
Effect of varying thresholds for myelin-weighted maps or functional delineations of hMT+. To test whether the degree of overlap between structural and functional area definitions were influenced by threshold selection, the thresholds for myelin-weighted maps based on the 7T MP2RAGE image and functional definitions of hMT+ were systematically changed. On the left, we show for the 2 hemispheres of 1 participant the effect of changing the myelin-weighted maps to a “very low threshold” (mean intensity), to a “low threshold” (mean intensity +0.5 SEM), and to a “high threshold” (mean intensity +1.5 SEM). These myelin-weighted maps were compared with the original functional definitions of hMT+ at *z* > 2.3. Functional definitions were also varied systematically for the same brain (right). We examined a “very low threshold” (*z* > 1.3), a “low threshold” (*z* > 1.8), and a “high threshold” (*z* > 2.8). The different area definitions of hMT+ obtained were then compared with the original myelin-weighted map at a threshold of mean intensity +1 SEM*.*

**Figure 9.**
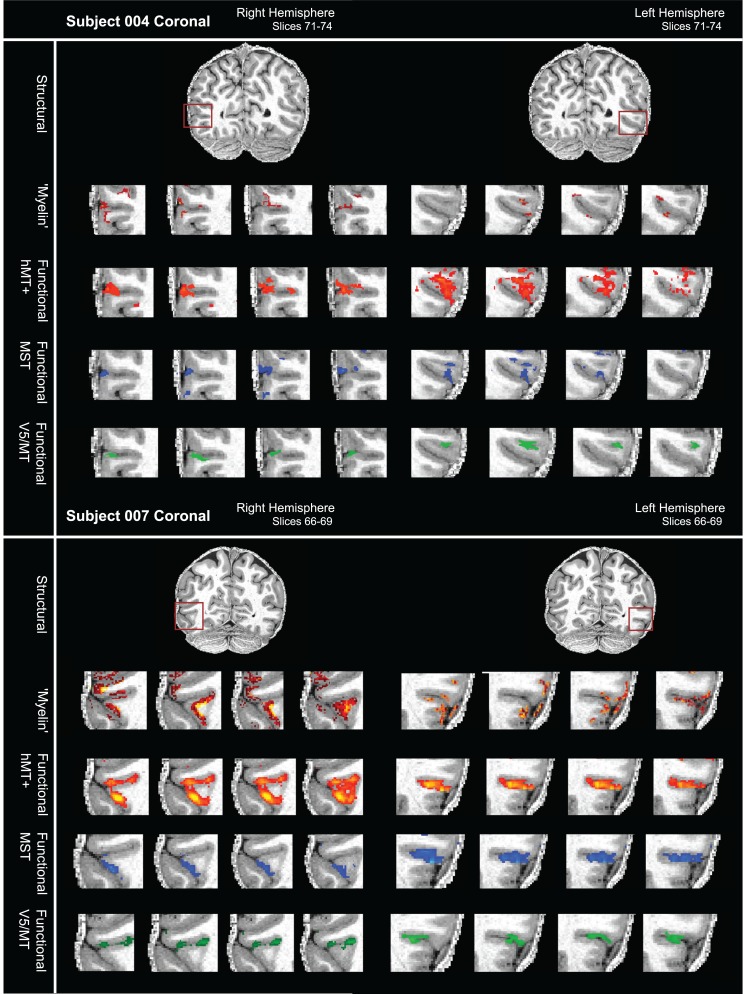
Variable overlap between myelin-weighted sMRI and functional localizers for hMT+, V5/MT, and MST in native space. (*A*) sMRI (7T MP2RAGE) and fMRI (7T) data are shown in a series of coronal slices (1 mm separation; left to right) for both hemispheres of participant 004. From top to bottom, the sequence of slices shows first the structural scan with the myelin-weighted map overlaid (red-yellow scale; threshold at mean intensity +1 SEM), then with functional activation for full field motion (red-yellow scale; threshold: *z* > 2.3; cluster threshold: *p* = 0.05), with functional activation for ipsilateral motion (blue; threshold: *z* > 2.3; cluster threshold: *p* = 0.05) and finally with the contralateral polar retinotopic activation (green). The red box on the structural image at the top indicates location of the visual motion area hMT+. Broadly, the regions of dense myelination are next to, and to varying degrees overlapping with, the region of functional activation for hMT+. Data are generally restricted to the cortical ribbon. (*B*) sMRI and fMRI data for a second participant (007), conventions as in (*A*). Regions of dense myelination are next to, and partly overlapping with, the regions of functional activation. Data are well restricted to the gray matter. See also Supplementary Figure S2 for further examples.

The quantitative analysis of the overlap between areas of high intensity in the structural images with functionally defined visual areas supports the view that parts of the region around hMT+ are heavily myelinated in individuals (Tables 4 and 5). But the degree of overlap of the myelin-weighted map derived from sMRI with functionally defined hMT+ and the exact location in relation to the functionally defined areas V5/MT and MST subdivisions varied from individual to individual. This result did not change when thresholds for myelin-weighted maps or functional delineations were systematically varied (Fig. 8). A further source of variability could be the conversion from native space to inflated surface. However, a qualitative comparison of the distribution of functional activations and myelin-weighted sMRI signal obtained at 7T in coronal sections showed similar results. There was considerable variability in the alignment between functional localizers for hMT+ and indication of high myelin-weighted sMRI signal (Fig. 9; see also Fig. 4 and Supplementary Fig. S2 for further examples).

This variability in the overlap between sMRI and fMRI definitions of hMT+ within individuals was mirrored by a considerable variability between participants in the cortical location of structurally and functionally defined hMT+. When 7T MP2RAGE myelin-weighted maps for hMT+ were projected on the standard brain and compared in native space, we found that mean pairwise overlap was only 6.9% (SD = ±5.2) for the left hemisphere and 5.9% (SD = ±4.7) for the right hemisphere. The pairwise overlap was similarly low for 3T *T*_1_w/*T*_2_w myelin-weighted maps (left hemisphere: 10.2%, SD = ±3.0; right hemisphere: 2.1%, SD = ±2.2) and for functionally defined hMT+ (left hemisphere: 13.6%, SD = ±7.6; right hemisphere: 11.8%, SD = ±7.6).

In contrast to the individual data, the quantitative analysis of the group average 7T MP2RAGE myelin data showed an almost exclusive correspondence with area V5/MT relative to MST (up to 58% for V5/MT vs. <2% for MST). However, the group average 3T *T*_1_w/*T*_2_w myelin data showed a large overlap with both V5/MT (up to 41%) and MST (up to 94%). To ensure that this difference was not driven by the myelin patterns of the 3 individuals who underwent both types of scans, we produced a separate group average 7T MP2RAGE image consisting of only the 6 hemispheres from 3 subjects who comprised the 3T *T*_1_w/*T*_2_w group average. This additional group average 7T MP2RAGE showed the same trend to predominant association of myelin-weighted signal with functional V5/MT as the full group average, indicating that the differences are more likely associated with the 2 different scanning protocols and not specifically related to these 3 subjects.

Overall, these results suggest that while the sMRI protocols used here are sensitive to structural differences associated with cortical myelin, the extent and location of myelination detected around functionally defined area hMT+ varies across individual humans and between individuals and the group average. Therefore, it is difficult to demarcate the borders of functional hMT+ based on the myelin-weighted maps.

## Discussion

This study provides the first systematic comparison of myelin-weighted cortical maps based on in vivo and postmortem sMRI with histology in individual primates, alongside a direct comparison of high-resolution anatomical and functional maps obtained from the same human brain. This approach is essential to evaluate imaging techniques and to understand the extent to which structural definitions of human cortical areas map onto regions of different functional organization. Our macaque data demonstrate that sMRI protocols can reliably detect a region of heavy myelination at the anatomical locations of the V5/MT-MST complex in individual hemispheres. The association of myelin-weighted sMRI maps with high-resolution functional maps of hMT+ in humans, however, showed considerable variation. Group average data sets did not capture adequately the myelin-weighted maps in individual brains, which showed a high degree of individual differences. We discuss potential sources of this variability, for example, lack of sensitivity of structural or functional methods used; alternatively, our MRI protocols might detect individual differences in myelination and in the relationship between myelination and functional areas in the human brain.

First, we consider the sensitivity and reliability of the myelin-weighted maps obtained with sMRI. Employed previously to assess myelin density in cortex, the two types of structural contrasts used in this study, 3T *T*_1_w/*T*_2_w and 7T MP2RAGE, produce images that are highly *T*_1_-weighted, but without the image intensity bias created by inhomogeneous transmit and receive RF profiles. The presence of myelin in the cortex causes a shortening of the *T*_1_ relaxation time, so methods that are sensitive to *T*_1_ should also reflect local myelin density (Barbier et al. 2002). The direct comparisons of histology and sMRI data from macaques demonstrates that sMRI is, in principle, sensitive to structural differences associated with cortical myelin in individuals and allows the generation of plausible myelin-weighted maps. In humans, both scan types showed generally high-intensity signals where expected from histology—around the central sulcus, striate cortex, and area hMT+ (Annese et al. 2005; Glasser and Van Essen 2011; Sereno et al. 2013). But sensitivity to dense myelin in different brain regions appeared to differ between the two types of structural scans. Macaque histology showed differences in the pattern of myelin across layers between areas identified as heavily myelinated (Fig. 1) (Lewis and Van Essen 2000), which could potentially affect sMRI measurements. A limitation of the human sMRI data—evident in our 7T data—was the confinement of much of the sMRI signal, indicating high myelin density near hMT+, to immediately above the border between white matter and gray matter. This might potentially limit the ability of sMRI to detect different patterns of myelination across the cortical layers. However, neither sMRI method used here in humans can provide fully quantitative *T*_1_ maps; from these data, it is unclear whether the different sMRI protocols reveal important differences in myelin contrast. In our human data, it may be that the differences in the *T*_2_ and *T*_2_* sensitivities of the methods make them more or less sensitive to tissue iron, local blood oxygenation, or local diffusion. Localization might therefore be affected by such differential sensitivities, rather than myelin directly. Further research is needed to understand which differences in sensitivity between scan protocols can be attributed to scanner strength (3T vs. 7T) and spatial resolution, and which might potentially be explained by different sMRI methods picking up distinct qualities of myelinated cortical regions. However, repeated MP2RAGE scans in the same subject yielded almost identical myelin maps demonstrating—as do the matching results from the macaque—the reliability of the sMRI data obtained.

Second, as in the Rhesus macaque, we expected to find a distinct region of heavy myelination corresponding to functional hMT+ in humans. Neurophysiology research over the last 4 decades has consistently linked a region of dense myelination with neural recordings from macaque visual area V5/MT (e.g., Dubner and Zeki 1971; Van Essen et al. 1981; Albright et al. 1984; Desimone and Ungerleider 1986; Maunsell and Van Essen 1987; Dodd et al. 2001). In our human data, the 7T MP2RAGE myelin-weighted map was associated with functional V5/MT for the same brains. Group averaged 3T *T*_1_w/*T*_2_w myelin-weighted maps covered much of functional hMT+, but especially MST, as reported previously (Glasser and Van Essen 2011; Abdollahi et al. 2014). A direct correspondence between the myelin-weighted map and the functional map is contradicted by the degree of variability we observe between individual human brains in the association of myelin signal with visual motion area hMT+ with either scanning protocol. This result is in line with previous studies comparing myelin maps and tonotopic maps in human auditory cortex, which also noted greater variation in the alignment of anatomical and functional maps in individuals compared with average data sets, but there are limits on the accuracy of these functional maps because they were acquired at lower resolution (Dick et al. 2012; De Martino et al. 2015).

Individual variation in grouped data sets seems to be erased by the central tendency of averages, even though cortical myelination may have a very variable pattern across individuals. But the crucial point remains, even when high-resolution sMRI and fMRI methods are applied to the same brain, the congruence of a region of dense myelin with a functional definition found at the group average level is not necessarily a good predictor of the precise pattern found at the level of the individual human brain.

Several reasons could account for the variation in individual human myeloarchitectural patterns and their association with functional hMT+:

1. variance generated by incorrect alignment of multiple different MRI images,
2. low sensitivity of fMRI methods employed, and
3. effects of actual structural differences in human brains.

MRI data require considerable processing, opening the door to potential error. Specifically, if the registration between our structural and functional maps were incorrect, it could account for some of the variation we see. However, each functional–structural registration was confirmed in FreeSurfer (via tkmedit). Furthermore, the consistency of myelin-weighted maps from 7T MP2RAGE images (which required no registration or averaging as the 2 echo times were interleaved) and 3T *T*_1_w/*T*_2_w images (which required multiple registrations and averaging) suggests that this was not an issue. Another potential source of noise in the comparison of structural and visual maps is the functional mapping of hMT+, V5/MT, and MST. This might vary because of differences in local vasculature, hemodynamic response efficiency, or orientation of the vasculature relative to the static magnetic field (B0). Such potential confounds have been extensively discussed in the literature (Logothetis and Wandell 2004). Another potential source of noise is the accuracy of the functional localizers used to identify hMT+, V5/MT, and MST. While we used visual stimuli widely accepted to identify these areas, these stimuli also activate additional motion-sensitive visual areas such as the kinetic-occipital area (Dupont et al. 1997; VanOostende et al. 1997; Larsson and Heeger 2006). In order to control for the accuracy of the functional mapping relative to sMRI maps, future experiments should directly compare sMRI and fMRI cortical maps in individual macaques.

One striking difference between Rhesus monkeys and humans lies in the complexity and variability of the sulcal pattern, which can be quantified across individual brains (Ono et al. 1990; Thompson et al. 1996; Segal and Petrides 2012). Although major human brain sulci, such as the central sulcus, tend to be consistent across individuals, there is significant variance between less prominent sulci. The sulcus commonly associated with area hMT+ (the posterior limb of the inferior temporal sulcus) is present in all typically developed human adults, but its position is highly variable. Sulci in the parieto-occipital region vary both anterior-posteriorly and vertically by up to 19 mm between individuals (Thompson et al. 1996). This variance, combined with the variable position of functional hMT+ relative to the sulcus (Wang et al. 2015), can mean that hMT+ is found across a sulcus, wholly on a gyrus, or entirely in a sulcus. For most of our subjects, functional hMT+ spanned a gyrus and a sulcus, but the sulcal extension varied between individuals. Sulcal location could affect the ability of sMRI to detect myelinated regions on a case-by-case basis, as gyrification of cortex can introduce variation in the voxel orientation relative to cortex and thus lead to partial volume effects (Clare and Bridge 2005).

In contrast, macaque area V5/MT sits reliably on the posterior bank of the STS and area MST on its anterior bank (Zeki 1974; Van Essen et al. 1981; Desimone and Ungerleider 1986), which might contribute to a less variable myelin-weighted map. However, the cortical thickness in humans and macaques is comparable and myelin-weighted maps based on in vivo sMRI in human and macaque were obtained at similar resolutions, making them similarly susceptible to image processing and partial volume effects. Nevertheless, histological sections, high-resolution postmortem and in vivo sMRI images show the same trends. To determine whether sMRI myelin detection is affected by sulcal position, location and extent of myelin-weighted maps could be correlated with anatomical location on the cortical surface, specifically comparing the sulcal banks, fundus, and gyral crowns in a larger data set. However, the appearance of anatomical myelin density might already be correlated with sulcal location, for instance with lower cortical layers stretched around the fundus appearing often less dense on histological sections. This could be one potential explanation for the individual differences in human myelin-weighted maps we observed.

It should also be recognized that the population of macaques in this study was most certainly drawn from a smaller genetic pool than our human participants. The macaques stem from the same breeding colony, which has been established for some years. In humans, for instance, it has been shown previously that quantity of gray matter across a range of cortical regions is more similar between twins than between unrelated individuals (Thompson et al. 2001). Larger population studies in humans that are underway may help to identify the reasons for the variability that we have demonstrated here.

Overall, our results show that sMRI methods can detect dense myelination in individuals. In humans, myelin-weighted maps vary considerably between individuals and myelin signals can “anchor” but not delineate functional hMT+ in individuals. Future research needs to distinguish potential sources for these individual differences in humans in order to determine how this type of myelin-weighted mapping can be used to delineate the borders of hMT+ and other functional areas. Our ability to replicate myelin maps with different methods in Rhesus macaques and within the same subject over time in humans suggests that these individual differences might represent structural differences between human brains, which could be exploited for research or in the clinic.

## Supplementary Material

Supplementary material can be found at: http://www.cercor.oxfordjournals.org/

## Funding

Biotechnology and Biological Sciences Research Council (UK) (BB/H016902/1); the Volkswagen Foundation (Az 85 060); and the Wellcome Trust (101092/Z/13/Z). IL held a Studentship from the Volkswagen Foundation. KK and HB held Royal Society University Research Fellowships.

## Supplementary Material

Supplementary Data
