# Supplementary material for: Individual Differences in the Alignment of Structural and Functional Markers of the V5/MT Complex in Primates
Source: Cereb Cortex. 2016 Sep 19;26(10):3928–44. doi: 10.1093/cercor/bhw180 (PMC5028002; doi:10.1093/cercor/bhw180)
Supplement: Supplementary Data [file supp_26_10_3928__index.html]

Individual Differences in the Alignment of Structural and Functional Markers of the V5/MT Complex in Primates — Individual Differences in the Alignment of Structural and Functional Markers of the V5/MT Complex in Primates — Individual Differences in the Alignment of Structural and Functional Markers of the V5/MT Complex in Primates — Supplementary Data 

# Individual Differences in the Alignment of Structural and Functional Markers of the V5/MT Complex in Primates

## Supplementary Data

Supplementary Data

- Supplementary Data - eps file
- Supplementary Data - eps file
- Supplementary Data - docx file
- Supplementary Data - eps file
