# Supplementary material for: Individual Differences in the Alignment of Structural and Functional Markers of the V5/MT Complex in Primates
Source: Cereb Cortex. 2016 Sep 19;26(10):3928–44. doi: 10.1093/cercor/bhw180 (PMC5028002; doi:10.1093/cercor/bhw180)
Supplement: Supplementary Data [file supp_bhw180_Supplementary_Figure_legends.docx]

**Supplementary Figure legends**

**Supplementary Figure S1.** *Slice-by-slice comparison of histological sections and matched sMRI for the Rhesus macaque.* **A.** Gallyas stained parasagittal sections from the left hemisphere of one macaque (M126L) were matched to the equivalent parasagittal sections obtained previously from a *post mortem* scan of the same intact, perfused brain and to MR images obtained *in vivo*. Histological sections are 50 μm thick; shown is a one in ten series with a separation of 500 μm between consecutive sections. Dense cortical myelin shows up as dark brown, particularly in the lower cortical layers in the histological sections, as dark areas relative to surrounding cortex in the T2-weighted MRI scans, and as lighter voxels for the T1w/T2w images. Yellow arrows indicate borders of dense myelination for extrastriate visual area V5/MT on the posterior bank of the Superior Temporal Sulcus (STS), and for parts of MST on the anterior bank for all three methods for an example section. Series orientation: lateral (L) to medial (M); section orientation: D - dorsal, V- Ventral, P - posterior, A - anterior. **B.** Gallyas stained parasagittal sections from the left hemisphere of one macaque (M127L) were matched to the equivalent parasagittal sections obtained previously from a *post mortem* scan of the same intact, perfused brain and to MR images obtained *in vivo*. Same histological and scanning protocol details as in 2A. Yellow arrows indicate borders of regions with dense myelination for extrastriate visual area V5/MT on the posterior bank of the STS, and for parts of MST on the anterior bank with all three methods for one example section. Conventions see S1A.

**Supplementary Figure S2.** *Variable overlap between myelin-weighted sMRI and functional localizers for hMT+, V5/MT and MST in native space* –additional participants. **A.-F**. sMRI (7T MP2RAGE) and fMRI (7T) data near hMT+ are shown in a series of coronal slices (1mm separation) for both hemispheres. This figure shows native space MRI data for six additional participants to those shown in Figure 9 and Figure 4 of the main paper (001, 003, 008 rh, 021, 022, 038). The sequence of images per row first show the structural scan with the myelin-weighted map overlaid (red-yellow scale; threshold: mean intensity +1 SEM), then functional activation for full field motion (red-yellow scale; threshold: z > 2.3; cluster threshold: p = 0.05), followed by the functional activation for ipsilateral motion (blue: threshold: z > 2.3; cluster threshold: p = 0.05) and finally, the polar retinotopic activation (green; response coherence > 0.25). The red box on the structural image in the top row indicates location of the visual motion area hMT+ for each participant. Data were generally restricted to the cortical ribbon, with variable overlap of the high myelin signal with functional localisers for the visual areas hMT+, V5/MT, and MST.
